# How distorted food prices discourage a healthy diet

**DOI:** 10.1126/sciadv.abi8807

**Published:** 2022-03-30

**Authors:** Roberto Pancrazi, Thijs van Rens, Marija Vukotić

**Affiliations:** 1 Department of Economics, University of Warwick, Coventry CV4 7AL, UK.; 2 Centre for Macroeconomics, London, UK.

## Abstract

Public policy making for the prevention of diet-related disease is impeded by a lack of evidence on whether poor diets are a matter of personal responsibility or a choice set narrowed by environmental conditions. An important element of the environment is market imperfections in food retail that distort prices. We use a rich dataset on quantities and prices of food purchases in the United States and a structural model of dietary choices to examine variation in diets across households that have different levels of income and live in different neighborhoods. We find that price distortions account for one-third of the gap between the recommended and actual intake of fruits and vegetables. A feasible fiscal intervention that remedies these distortions makes all consumers better off.

## Teaser

A subsidy on fruits and vegetables that fixes price distortions would improve diets and could make all consumers better off.

## INTRODUCTION

Diet-related disease leads to more preventable deaths in the United States than any other risk factor. In 2005, obesity and overweight on their own were the third largest preventable cause of deaths (11%) after smoking (24%) and high blood pressure (20%). When other diet-related risk factors are included, that contribution grows to 28% (*1*). The situation in most other developed economies is similar. In the United Kingdom, for instance, the chief executive of NHS England warned that “Obesity is the new smoking,” with obesity and overweight about to overtake smoking as the biggest cause of cancer (*2*). This is a relatively new public health problem. Since 1980, obesity rates have increased sharply (*3*), taking a staggering economic and human toll (*4*). This is even more true recently, with obesity emerging as a major risk factor for coronavirus disease 2019 (COVID-19) (*5*–*7*). Given these far-reaching implications of dietary choices, here, we ask what are the most important determinants of our diets, and whether there is a role for policy to improve what we eat.

Understanding the role that public policy can play depends on knowing to what extent poor diets are a matter of personal responsibility, and to what extent they are due to a choice set narrowed by environmental conditions. If poor diets are mostly an “environmental curse” (*8*), then policy interventions that place most responsibility with the individual consumer will be ineffective (*9*). Likewise, if diets are mostly determined by individual preferences, then the role for policy intervention is limited and well-intended interventions could do more harm than good. Here, we aim to quantify the role of market imperfections in food retailing that distort relative food prices. The specific imperfection that we focus on is the fixed costs in the supply of healthy food, which are much larger for healthy than for unhealthy food products. The price distortions brought about by this imperfection represent a specific set of environmental distortions that justify government intervention to improve dietary choices.

Price distortions are likely to be an important aspect of the environmental determinants of diet. A large literature in public health explores the effect of “obesogenic environments” (*10*), a term first coined by public health expert Boyd Swinburn (*11*). The environment may matter for obesity because of its effect on inactivity (opportunities for exercise and active transport) or on diet, through access to healthy food, advertising, labeling, and food prices. There is a notable lack of evidence in the literature on the potential effect of prices. Cawley (*12*) summarizes the problem as follows: “A large number of papers have regressed obesity or BMI on local food prices; the limitation of this approach is that variation in local food prices may be due to differences in demand over geography and changes in demand over time and thus the estimates of the impact of local food prices on weight may be biased.” (p. 248). “Economic research with good identification strategies often is unable to reject the null hypothesis of no effect from many possible causes, such as food prices, ...” (p. 261).

It seems plausible that the deteriorating quality of people’s diets and the resulting obesity epidemic are at least partly due to prices. Aggregate prices for healthy food groups, in particular for fruits and vegetables (*13*), have increased relative to prices of unhealthy food and drink (*14*). Since 1980, inflation-adjusted prices for fresh fruits and fresh vegetables have grown by 29 and 49% more than average food prices, respectively, according to data from the UK Bureau of Labor Statistics. The timing in these relative price changes roughly lines up with the start of the obesity epidemic in the United States, which is generally thought to have begun around 1980 (*3*). However, there is a limit to what can be learned from the time series, and it is almost impossible to disentangle different possible explanations using aggregate data alone. Thus, this paper attempts to better understand the determinants of dietary choices by studying variation in diet across households of different income levels and in different locations (counties).

We first conduct a reduced-form analysis of quantities and prices of food purchased by U.S. households, using the Nielsen Consumer Panel (Homescan) data. The data show clear income gradients in the consumption of fruits and vegetables, as a proxy for healthy food more generally: The share of fruits and vegetables in total food consumption strongly increases with both household income and average income in the county where households reside. The corresponding gradients in prices of fruits and vegetables shed light on what explains this dietary inequality. Prices of fruits and vegetables, relative to the prices of other food items, increase with household income but decrease with the average income level of the county for counties that have average incomes below the national average. Although versions of these facts have recently been documented by others as well (*15*), we are the first to argue that the two facts that we document, taken together, indicate that both demand and supply factors are important determinants of diets. If differences in diet were driven exclusively by demand shifters (e.g., preference heterogeneity), then differences across households would trace out the supply curve, and we would expect to see a positive correlation between relative consumption and relative prices.

We then use a structural model to quantify the relative importance of demand and supply determinants of diets. The crucial element of the model is the retail technology for food. We assume that there are fixed costs associated with retailing food. We think of these as the costs associated with operating a food store and its supply chains. The fixed costs generate a downward-sloping supply curve: The more a particular type of food is sold in a county, the cheaper it is. This is because the fixed costs can be spread out over a larger amount of sales. This model predicts that relative prices are inefficiently high for foods with large fixed costs of supply, especially for foods in low demand. We make the model flexible enough to match the data by assuming that preference parameters may vary exogenously with income. This feature of the model allows preferences to determine diets. We then estimate the model to let the data inform us about the relative importance of the various determinants of dietary inequality.

We are able to separate demand and supply factors by making the identifying assumption that households living and shopping in the same county face the same menu of prices. This assumption allows us to use variation in prices and quantities across households within a county to estimate the contributions of heterogeneity in preferences and the relative demand elasticity (demand shifters). We then use prices and quantities across counties to estimate the fixed costs of supplying food (supply shifters) as the remaining variation that is unexplained by preferences. We implement the estimation using nonlinear simulated method of moments on the same data that we used for the reduced-form analysis.

We find evidence for distortions in the relative price of healthy food, driven by large fixed costs in the supply of fruits and vegetables. Our estimates suggest that these distortions result in a relative price that is about 40% higher than if markets were efficient. This price distortion then translates in relative consumption of fruits and vegetables that is about 15% too low. This effect of the food environment on diets is smaller than the effect of preferences, but it is economically and statistically significant and large. Moreover, our focus on prices excludes many other externalities that may distort dietary choices. Thus, our estimates should be interpreted as a lower bound for the role of the food environment in discouraging a healthy diet.

Dietary inequality due to price distortions is inefficient. Therefore, there is a role for government intervention to improve diets. Using our structural model, we analyze the counterfactual effect of various policies. Although any remedy for distortions in food prices will generally affect the distribution of resources across consumers, we show that it is theoretically possible to design an intervention that replicates the first-best allocation and makes all consumers strictly better off. We then discuss a feasible tax and subsidy policy that closely approximates this compensated Pareto-improving intervention.

Two recent papers in the economics literature ask questions that are closely related to the subject of this study, use the same data, but reach conclusions that appear to be quite different from ours. Allcott *et al*. (*16*) show that households of different income levels that buy food in the same store nevertheless make different healthy and unhealthy food choices, illustrating the role of preferences for diets. They also show that households that move to a healthier environment for exogenous reasons do not (immediately) change their diets very much. In their interpretation, these findings cast doubt on the idea that the environment plays an important role in diets. Amano-Patiño (*15*) estimates a demand system for food products on household panel data and similarly finds that the role of prices in explaining differences in diet across households of different income levels is very limited. Similarly, DellaVigna and Gentzkow (*17*) find that the variation in prices across most U.S. food (as well as drugstore and mass merchandise) chains is rather small, regardless of consumer income. Our data confirm the findings in these studies, and we find that the variation in food prices and the effect of distortions in the food environment on dietary inequality are small. Nevertheless, even these small differences in cross-sectional dieting patterns and in prices reveal an important effect of distorted food prices on diets of all households, regardless of their income. In the terminology of a randomized experiment, we use the (small) differences between poor households (the treatment group) and rich households (the control group) to estimate the effect of food prices on diets. These estimates then indicate that the effect of prices is large, but affects both groups of consumers, with only small differences in the treatment between treatment and control group.

The remainder of this paper is organized as follows: We first introduce the Nielsen Homescan data and document some patterns in dietary inequality that lead us to conclude that healthy or unhealthy dietary choices are not determined exclusively by preferences. To quantify the role of the food environment, we need to put some structure on the data. We then present a simple and flexible structural model of consumers’ choices between healthy and unhealthy food, as well as a discussion of how we identify and estimate this model. Last, we turn to results and show that distorted relative food prices due to high fixed costs in the supply lead to inefficient underconsumption of fruits and vegetables by 14 to 15.5%. Fixing this distortion would close between a quarter and almost all of the gap between actual and recommended intakes of fruits and vegetables. We also show that it is theoretically possible for the government to intervene in such a way that makes consumers of all income levels and in all locations strictly better off, and that such an intervention can be closely replicated by a subsidy on fruit and vegetable consumption funded through a proportional income tax.

## RESULTS

### Income gradients in fruit and vegetable prices

In this section, we document some patterns in food purchases by income. We use the Nielsen Consumer Panel (Homescan) dataset. It contains detailed information about quantities and prices of food purchases over the 2004–2014 period by about 60,000 households that were given a barcode scanner to record their purchases of 1.7 million distinct food products. Although Nielsen Retail Scanner arguably contains better-quality information on county-level prices and consumption levels, we use the Homescan data because, unlike the Retail data, these also include purchases of so-called random-weight products that are weighed in store and therefore do not have a Universal Product Code (UPC). Given the focus of our analysis on fruits and vegetables, omitting information on fresh produce without UPCs would be undesirable.

We use a two-step procedure to aggregate product data to product groups. First, we calculate household-level aggregate quantities for product groups *Q_jh_* as the weighted sum of the quantities *q_ijh_* of all products in that group, using as weights the average price of each product across households ${\bar{p}}_{\mathrm{ij}}$, $Q_{\mathrm{jh}}=\sum_{i\in j}\frac{{\bar{p}}_{\mathrm{ij}}}{{\tilde{P}}_{j}}q_{\mathrm{ijh}}$. Second, we calculate household-level aggregate prices for product groups by dividing household-level expenditures by the household-level aggregate quantity. This approach to aggregating quantities and prices of food products is based on how the Bureau of Labor Statistics aggregates product prices and quantities when measuring inflation, and provides an alternative to the nutrition-based approach used, e.g., in (*15*) and (*16*). The advantage is that we use market prices of different products, by revealed preference, as measures of the value of each product to consumers. A more detailed description of the data and of the aggregation of quantities and prices is provided in Materials and Methods.

We start by documenting how purchases of different types of food vary with income. Household income is included in the Nielsen data, and we obtain average income in the location of residence for each household by matching their ZIP code to income data from the Census. We smooth the data by projecting purchase quantities and prices on a bivariate 10th-order polynomial in household and location income, and then aggregate to 5-percentile bins in these variables. In some specifications, we also correct for household composition and demographics by including second-order polynomials in household size, age, and years of schooling (normalized at the sample mean) and dummies for whether two adults are present in the household and for household heads that are female, African American, Asian, other race, Hispanic, part-time employed, or nonemployed in the regression.

**Fig. 1.**
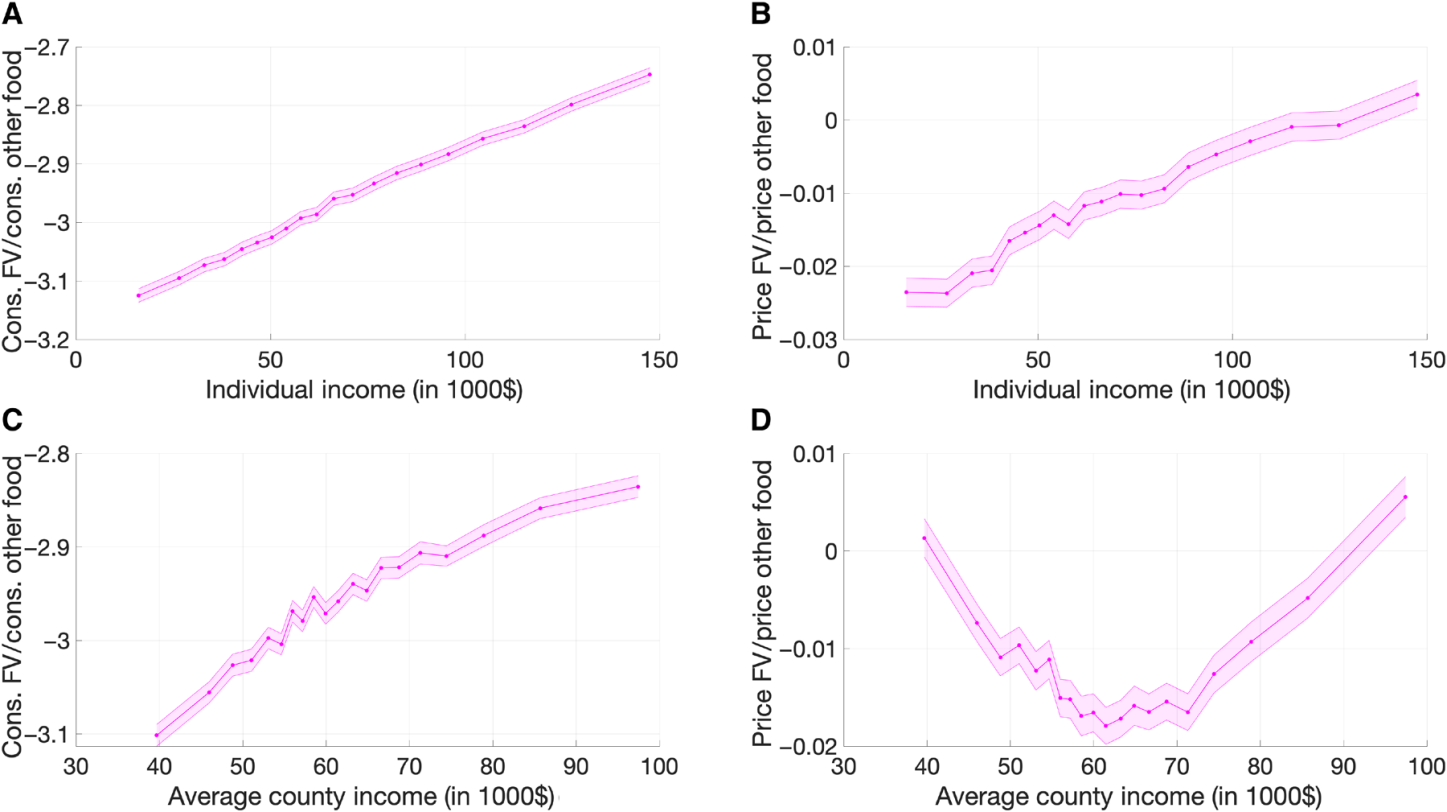
Relative consumption and prices. (**A**) Consumption of fruits and vegetables (FV) as a fraction of total food consumption against individual income. (**B**) Relative price of FV against individual income. (**C**) Consumption of FV as a fraction of total food consumption against income across counties. (**D**) Relative price of FV against income across counties. The shaded areas represent a two-standard error confidence band. Each point is a 5-percentile bin.

Poorer households consume much less fresh produce than richer households (see Fig. 1A). The diet of the poorest 5% of households contains around 40% fewer fruits and vegetables than the diet of the richest 5%, a finding robust to broader definitions of fruits and vegetables. This difference is large compared to the overall underconsumption of fruits and vegetables relative to the recommended intake of 40 to 60% above current average intakes [see (*18*), figures 2.3 and 2.4]. Vegetables and fruits (and whole grains) are the most important two (three) of the six components of a healthy eating pattern, and they are also the food groups for which current intakes are furthest below recommended levels [see (*18*), chap. 1, p. 15]. This paper aims to explore and understand causes of this underconsumption.

What drives the difference in diet between poor and rich households? Clearly, there is a role for demand factors. People have different preferences for different foods, and these preferences may be correlated with other characteristics and income. More generally, “preferences” may be shaped by education, parental income and education, and socioeconomic status—all of which are correlated with income as well. On the other hand, it is equally clear that supply factors may matter, too. Much has been written about obesogenic environments, which may feature limited access to healthy food, abundant advertising and marketing for unhealthy food, misleading food labels, and distorted relative prices of healthy and unhealthy food options. Here, we focus exclusively on the role of price distortions. Hence, our estimates should be seen as a lower bound for the effect of environmental constraints on diets.

Comparing prices paid for fruits and vegetables by income levels, as in Fig. 1B, reveals the importance of preferences. Richer households not only consume a higher fraction of their diet in the form of fruits and vegetables but also pay relatively more for these healthy foods. Thus, it seems that their higher demand is the driving force that pushes up the prices. However, household-level purchase prices are not a good measure of market prices, because the household-level price of the basket of fruits and vegetables is determined in large part by the household’s choice about which products from that basket to purchase. Because richer households will tend to buy higher-quality or more desirable, more expensive types of fruits and vegetables, household-level prices will exaggerate the role of demand factors.

Thus, a better measure of environmental constraints is the relative price of healthy food in the location where the household lives and shops for food. Figure 1 (C and D) shows differences in relative consumption and prices of fruits and vegetables across counties. The rationale for choosing a county as the geographic unit is twofold. First, choosing a relatively large unit implies that any additional cost to source food within a location would be captured by preferences so that the role of market imperfections that we estimate is a lower bound for the importance of supply determinants of diets. Second, while we observe ZIP codes and Census Tracts in our data, we do not have enough households to work at this level of disaggregation and many ZIP codes have zero or one respondent only.

The inequality in diet between poor and rich households carries over to inequality in diet between poor and rich counties. While the underconsumption of fruits and vegetables in the poorest 5% of counties is about 25% larger than in the richest 5%, compared to a difference of 40% between the poorest and richest 5% of households, differences in income are also smaller across counties, and the gradient of underconsumption of fruits and vegetables with income is even higher across counties than across households. However, geographic variation in relative prices paints a very different picture. Focusing on counties with below-average income levels, households in poorer counties face higher relative prices of fruits and vegetables. This finding underlies our main conclusion that market imperfections play a role in skewing the costs of healthy food, negatively affecting dietary choices. It is not possible to reconcile the negative correlation between relative quantities and prices of healthy food with a standard upward-sloping supply curve that is traced out by differences in demand factors.

We conclude that there must be a role for the environment in determining diets, at least in counties with below-average income levels. The next step is to quantify the role of these supply factors.

### Quantifying price distortions in the food supply

Estimating the relative importance of preferences and environmental constraints for diets requires that we impose a bit of structure on the data. We assume that dietary choices of household *i* in location *j* are made according to a standard economic model of consumption choice, maximizing a utility function over the consumption of healthy food *C*_*H*,*ij*_ and unhealthy food *C*_*U*,*ij*_, subject to a budget constraint. Further details are described in Materials and Methods.

The model is deliberately very simple. Its strength is that it is very flexible so that we impose no more than the minimum structure needed to quantity the role of supply factors. In particular, we allow exogenous parameter heterogeneity in the utility function over healthy and unhealthy food, which may be correlated with income (nonhomothetic preferences), and which will capture all demand-related determinants of diets.

Environmental constraints affect households’ dietary choices through prices *P*_*H*,*ij*_ and *P*_*U*,*ij*_, for healthy and unhealthy food, respectively, which are set by local food retailers in each location. Retailers buy food from a wholesaler so that the marginal cost *κ_H_*(*y_ij_*) of healthy and *κ_U_*(*y_ij_*) of unhealthy food of the same quality is the same in all locations, although this cost may depend on household income *y_ij_* because households have a choice over the quality of food items they consume. This exogenous dependence of the marginal costs on income is a second channel through which preferences may affect dietary choices.

In addition to these marginal costs, retailers face fixed costs $K_{H}({\bar{y}}_{j})$ or $K_{U}({\bar{y}}_{j})$ for supplying healthy or unhealthy food in a particular location. These fixed costs are allowed to vary across locations and may depend exogenously on average household income in location *j*, ${\bar{y}}_{j}$. We assume that there is free entry of food stores in each location. The fixed costs generate increasing returns to food supply so that, in equilibrium, there will be a single store for each food type in each location. However, the threat of entry implies that the local monopoly is only sustainable if retailers make zero profits. This assumption is consistent with the evidence in (*19*), who show that firms set prices with a markup over average rather than marginal costs. Under these assumptions, prices are set just high enough to recover fixed costs, which leads to the following pricing conditions

$$P_{H,\mathrm{ij}}=\frac{K_{H}({\bar{y}}_{j})}{{\bar{C}}_{H,j}}+\kappa_{H}(y_{\mathrm{ij}})$$ (1)

$$P_{U,\mathrm{ij}}=\frac{K_{U}({\bar{y}}_{j})}{{\bar{C}}_{U,j}}+\kappa_{U}(y_{\mathrm{ij}})$$ (2)

where ${\bar{C}}_{H,j}=\sum_{i\in j}C_{H,\mathrm{ij}}$ and ${\bar{C}}_{U,j}=\sum_{i\in j}C_{U,\mathrm{ij}}$ denote the total consumption in location *j* of healthy and unhealthy food, respectively.

Our model allows a role of preferences as well as supply factors as drivers of dietary inequality. Preferences broadly defined generate differences in diet across households with different income levels for two reasons: first, because households of different income have different preference for healthy food and, second, because households with different incomes have different preference for quality. Both effects lead to differences in prices. Inequality in diets that is driven by these two channels is efficient in the model, and there is no role for government intervention. But environmental constraints may matter as well. Households that live in a location where there is low demand for healthy food will face higher relative prices for healthy food, because the fixed costs have to be distributed over a smaller base of aggregate sales of healthy food. Inequality in diets originating from this effect is inefficient. The externality is a monopoly distortion, which creates a coordination problem among consumers. If the government were able to coordinate all households in a location to buy more healthy food, then these households would all benefit from lower prices for healthy food. Given estimates for the parameters, we can use the model to quantify the relative importance of each of these channels.

We estimate the parameters of our model by matching the model predictions to the patterns in dietary inequality documented above. Our identifying assumption is the following: While individual household demand depends on household-specific prices, prices in the supply curve do not depend on individual household demand, but on aggregate consumption in the location only (see Eqs. 1 and 2). This assumption allows us to solve the identification problem by using variation in quantities and prices across households both within and across locations. In Materials and Methods, we formally describe the identification of the model. In short, the intuition for our identification strategy is that households that live and therefore shop in the same location face the same prices. Therefore, any differences in the amount of healthy and unhealthy food they purchase, and the prices they pay for these foods, must be due to their preferences. Having identified the role of preferences from variation within locations, we can then use the variation across locations to identify the role of exogenous price shifters, too. The estimation of the model is implemented through nonlinear simulated method of moments.

**Fig. 2.**
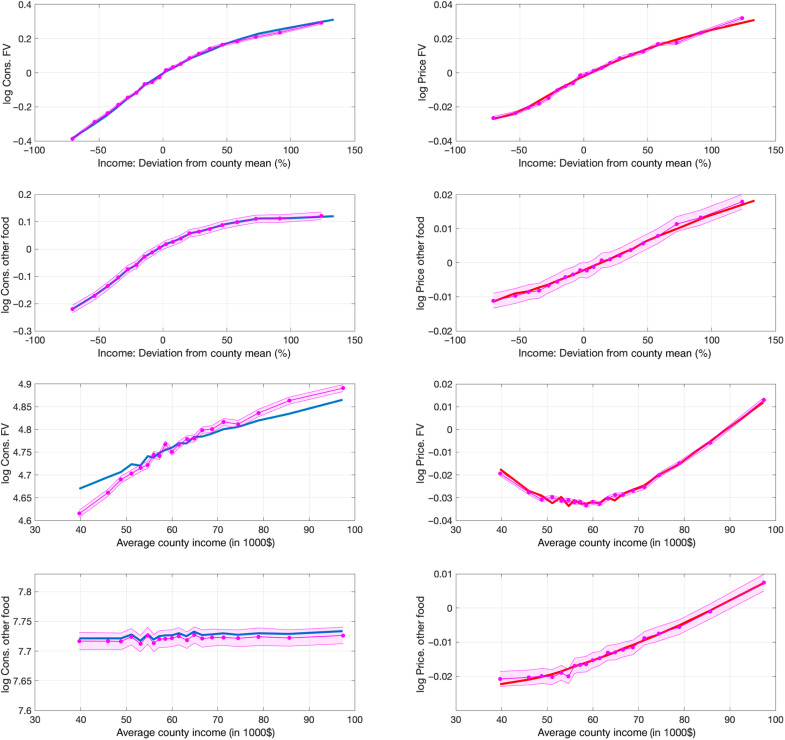
Model and data. This figure displays the model fit. The left column plots consumption levels, and the right column plots price levels. The first row displays consumption and prices of fruits and vegetables against income in deviations from the county mean. The second row displays consumption and prices of all other food against income in deviations from the county mean. The third row displays consumption and prices of fruits and vegetables against county income. The forth row displays consumption and prices of all other food against county income. The purple dotted line plots the data moments with the two–standard error confidence bands displayed as the shaded area, while the blue solid line plots the model moments. Each point is a 5-percentile bin.

Figure 2 shows the model fit. The purple dotted lines display the data moments, with the associated shaded two–standard error confidence bands, while the solid blue and red lines display the model-implied moments for consumption and price, respectively. The model is flexible enough to match almost perfectly the variation of consumption of healthy food (fruits and vegetables), consumption of unhealthy food (all other food), and their prices with income—both household income in deviation from the county average and county income.

**Fig. 3.**
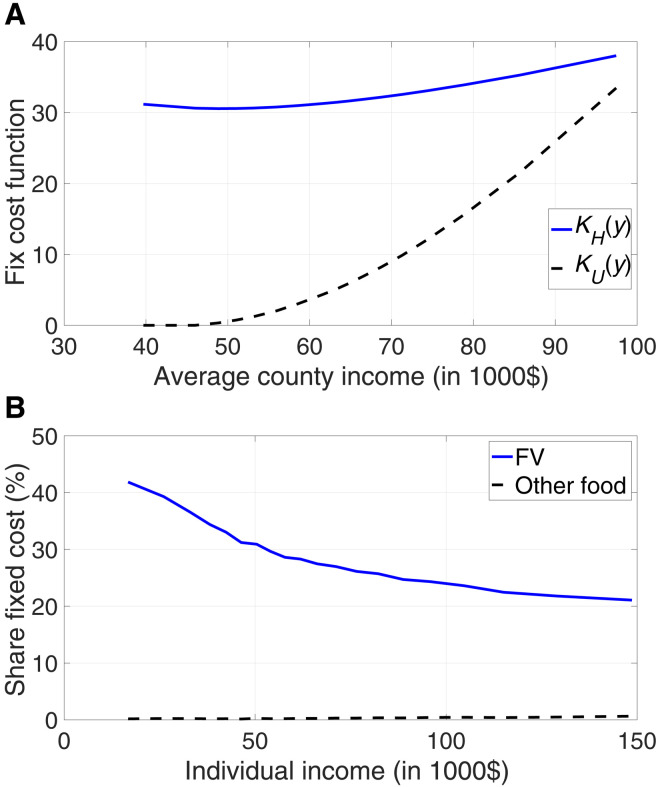
Fixed costs. (**A**) Estimated fixed cost as a function of county income. The solid blue line displays fixed cost in the healthy food sector, *K_H_*(*y*), and the dashed black line displays fixed cost in the other food sector, *K_U_*(*y*). (**B**) Share of the price due to fixed cost, in percent, as a function of individual income and computed as $\left(K_{z}({\bar{y}}_{j})/{\bar{C}}_{z,j}\right)$/*P*_*z*,*ij*_, with *z* ∈ {*H*, *U*}.

The estimated fixed costs in the supply of healthy and unhealthy food are shown in Fig. 3. The first panel (Fig. 3A) shows the estimated values of the fixed costs and how they vary with county income. Both fixed costs increase with average income in the county. This is what we would expect because retail prices and congestion would tend to make running a store in richer, more urban counties more costly.

The level of the fixed costs in healthy and unhealthy food cannot be easily compared, because total expenditures on the two types of food (fruits and vegetables versus all other food) are very different. Therefore, the second panel (Fig. 3B) plots the fixed costs again, but as a share of the prices of healthy and unhealthy food against individual income. This panel provides a measure of the magnitude of the market inefficiency for households of different income levels. As the share of fixed costs in the price approaches zero, the price approaches marginal costs and, as a result, the allocation is efficient.

The main finding from the parameter estimates is that the fixed costs for supplying fruits and vegetables are much higher than the fixed costs for supplying other food. For fruits and vegetables, fixed costs account for 20 to 43% of the price, whereas that same percentage is negligible (less than 1%) for other foods. This result is in line with the study of ver Ploeg *et al.* (*20*), who emphasize that perishable foods entail much higher fixed costs that are hard to spread without higher volume of sales or higher prices. This finding is important, because it implies that the market for healthy food is much more distorted than that for unhealthy food. Fruit and vegetable prices are in (large) part the result of a monopoly distortion that drives up prices where demand is relatively low, i.e., in poorer counties. The same is not true for other food, which is priced at very close to marginal cost. In this section, we developed a structural model that provides a plausible description of the variation in consumption and prices of healthy and unhealthy food across households and locations. We also estimated this model and showed that it fits the data well. We now use the model for counterfactual analysis.

### Interventions to improve diets

How much do distortions in food prices affect diets? Is there a role for policy to improve what we eat? We address these questions by comparing the relative consumption of fruits and vegetables in equilibrium to a diet determined by a benevolent social planner, who allocates the “first-best” or efficient consumption for households. The planner is constrained by the retail technology and the distribution of consumers over locations. She must also respect the aggregate resource constraint. The planner’s optimization problem is described in Materials and Methods. In the efficient allocation, the relative consumption of healthy food depends only on preferences over diet and marginal costs. In equilibrium, dietary choices are distorted because prices are not equal to marginal costs.

**Fig. 4.**
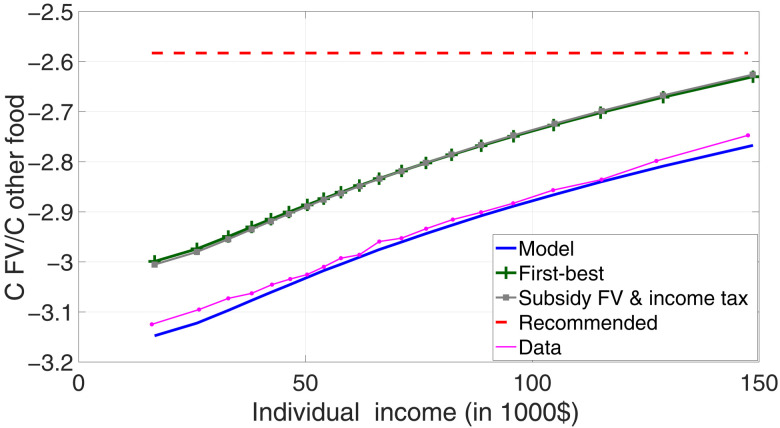
Subsidy and taxes: Consumption allocation. This figure displays the relative consumption of fruits and vegetables, against individual income, in the data (purple dotted line), in the estimated model (solid blue line), in the Pareto-efficient allocation (green crossed line), and in the model with fruit and vegetable subsidy financed by an income tax (gray squared line). Each point is a 5-percentile bin.

Figure 4 shows the relative consumption of fruits and vegetables in the first-best allocation (green crossed line), as well as in the equilibrium allocation estimated by the model (solid blue line) and its data counterpart (dotted purple line). For comparison, we have also plotted the recommended intake of fruits and vegetables from (*18*); this level is at least 40% above average current intakes (red dashed line). Comparing the first-best to the equilibrium allocation, consumption of fruits and vegetables is 14 to 15.5% lower than is efficient. This is due to price distortions, which are themselves due to supply factors. This effect is roughly constant for households of all income levels and across counties of different average income levels. Price distortions have almost no impact on dietary inequality, which explains why the difference-in-differences analysis in (*16*) that compared poorer to richer households revealed only a negligible role for prices.

In contrast, the effect of price distortions on diets is large. On average, these distortions are responsible for about one-third of the gap between the actual and recommended intakes of fruits and vegetables—ranging from almost a quarter of the gap for the poorest households to almost the entire gap for the richest 5% of households. It is important to reiterate that the role of supply factors we estimated is a lower bound because we consider only one inefficiency: the effect of fixed costs in the supply of healthy food. Other externalities are likely to be important. For example, consumers may not fully take into account the health effects on their diet, either because boundedly rational consumers discount the long-term effects of their dietary choices (*21*, *22*) or because of moral hazard if consumers are insured for their health care costs. Our model does not include these other externalities, and as result, their effect will therefore be attributed to preferences. We deliberately focus on a narrow set of supply factors to strengthen our argument: Even if we restrict the analysis to evident price distortions, there is a case for government intervention in the food retail environment.

The first-best allocation is a theoretical concept. We therefore explore the question of efficient allocation in practice. How close can a simple fiscal intervention come to achieving an efficient allocation? We examine a measure that can be feasibly implemented in reality: a flat-rate subsidy on fruits and vegetables. We look at the effects of this subsidy, which we posit would be funded by a progressive increase in income taxes to offset the unintentional regressive effects of the subsidy that could result because richer households consume more fruits and vegetables.

Let τ*_H_* denote the subsidy so that consumers face a price (1 − τ*_H_*)*P_H_* for fruits and vegetables. Let τ_*y*,*ij*_ be the income tax faced by the household *i*, *j*. The income tax is assumed to be proportional, i.e., τ_*y*,*ij*_ = ϕ*y_ij_*. We require that this tax and subsidy policy is neutral to the government budget, which implies that ∑*_ij_*τ*_H_P*_*H*,*ij*_ = ∑*_ij_*ϕ*y_ij_*. We then look for parameters τ*_H_* and ϕ that minimize the sum of squared deviations of relative consumption from the Pareto-improving efficient allocation. Total food expenditure will be affected by an income tax increase but is exogenous in our model. Therefore, we estimate an Engel curve for food, *m_ij_* = *f*(*y_ij_*), using a fixed-effects regression of a fourth-order polynomial in income with location-specific intercepts. We then use the estimated Engel curve to calculate the change in *m_ij_* due to the change in after-tax income (1 − τ_*y*,*ij*_)*y_ij_*. These calculations are described in more detail in Materials and Methods.

The gray squared line in Fig. 4 shows the consumption allocation with a subsidy on fruits and vegetables funded through an income tax increase. This simple fiscal intervention closely replicates the Pareto-improving efficient allocation (green crossed line). The required subsidy is 24.7%, and to fund this subsidy, the income tax rate needs to be increased only slightly, by 0.0482% points.

## DISCUSSION

This paper explores the extent to which dietary choices are influenced by the “environment” or by preferences. From a policy perspective, the distinction is important. If diets are mostly determined by external factors, then there is a stronger case for interventions. We focus exclusively on distortions in the relative price of healthy versus unhealthy food, with the aim of establishing a lower bound on the role of the environment. Other possible distortions, for instance, because consumers fail to (fully) internalize the health effects of their diet, are outside the scope of this paper.

We find that relative prices of healthy food are severely distorted. Fruits and vegetables are 40% more expensive than is efficient. We show that these price distortions lead to inefficient underconsumption of fruits and vegetables by 15%, accounting for about a third of the gap between actual and recommended intakes. By contrast, the effect of price distortions on differences in diet across households of different income levels is limited. This explains the contrast between our findings and those of recent previous studies.

We evaluate different interventions that could be used to improve diets. As a starting point, we calculate the first-best levels of healthy and unhealthy food consumption by all households as the allocation that a benevolent social planner would choose. We show that it is theoretically possible to achieve efficient relative price levels that support this first-best allocation as an equilibrium, without making any consumer worse off. This first-best allocation is just a theoretical concept: To achieve it in the real world, the government would have to give a subsidy on healthy food and levy a tax on unhealthy food that are specific to each individual household. However, we also show that a universal subsidy on fruits and vegetables, financed by an incremental increase in the income tax, comes very close to replicating the first-best allocation. We document that this simple fiscal intervention results in substantial overall welfare gains and in an acceptable distribution of these gains.

Our findings have obvious and clear policy implications. The inefficient underconsumption of healthy food due to price distortions implies that there really is no reason to not subsidize fruits and vegetables. Such a subsidy will make society as a whole better off, and by financing the subsidy appropriately (progressively), the policy maker can ensure that these gains are distributed in a fair manner across households of different income levels. Our estimates indicate that the subsidy on fruits and vegetables should be substantial, around 25%, to achieve the efficient allocation. The optimal subsidy is likely to be even higher, because our model does not take into account additional benefits of healthier diets like better health and lower health care costs. However, since our estimates are to some extent sensitive to assumptions, a cautious approach would be to start with a lower subsidy and evaluate its effects periodically also because, from a political perspective, it is often easier to raise or lower an existing subsidy (or tax) than to introduce a new one. As part of the periodic evaluations, the government should also consider which other food groups it may want to subsidize or tax, and it may make sense to consider such proposals in the context of a wider review of the sales tax or value-added tax (VAT).

## MATERIALS AND METHODS

### Data

As explained in the main text of our article, we use the Nielsen Consumer Panel (Homescan) dataset. The dataset is collected by Nielsen and distributed for academic use by the Kilts Center for Marketing at Chicago Booth. Participating households are given a barcode scanner and are instructed to scan their food purchases at home and enter quantity purchased and expenditure for each item so that unit prices can be obtained simply by dividing expenditure by quantity. Basic demographic information about the household is recorded as well. For this study, demographic information includes household income and place of residence (ZIP code).

Purchase information is available for about 60,000 households. Each household records on average 172 shopping trips and, on each given trip, records on average seven product purchases with distinct UPC. The data are highly disaggregated by product, because different varieties, different brands, and different package sizes are all coded as different UPCs, resulting in 1.7 million distinct food products. The complete dataset has around 50 million observations (product purchases) per year, for 10 years from 2004 to 2014.

A problem arises with random-weight items, foods that consumers purchase in any quantity they desire and are charged for by weight or quantity. This problem affects mostly fresh produce, but also unprepared meat, poultry, and seafood; bread and baked goods; deli counter items like cheese, deli meat, prepared salads, and foods; bread and baked goods; coffee; and dried vegetables and grains. In the dataset, these purchases are recorded as “magnet data” with a generated UPC that is much more aggregated than an actual UPC. In the first years of the sample, magnet data are only recorded as such. From 2007, the “product group” of these magnet data is also recorded. Product groups are 64 groups of similar UPC-level products, defined by Nielsen, which each constitute up to 4.1% of the average household’s food expenditures. Examples of product groups, ordered by expenditure share, are “bread and baked goods,” “snacks,” “packaged meats—deli,” “fresh produce,” “cheese,” “prepared foods—frozen,” “milk,” “carbonated beverages,” “candy,” and “juice drinks—canned and bottled.” Since fresh produce is particularly important for this study, we only use data for the 2007–2010 period, and we aggregate products to the product group level.

Household income is provided in the Nielsen Homescan data. We add average income in the location of residence for each household by matching their ZIP code to income data from the Census and then aggregating to the county level. There are 3007 counties in the United States so that we have on average 20 households in a county. The choice of a county as geographical unit depends on the fact that the number of ZIP codes is too large compared to the number of households in our dataset, which results in too few observations in each ZIP code to be able to document the relation between food purchases and income at this level. Household income is coded as a categorical variable, which we make continuous by assigning households an income level corresponding to the midpoint of the income range represented by the income category they belong to. Household income then ranges from $2500 to $120,000 per year in 16 categories.

### Aggregating quantities and prices

Our dataset contains detailed information about prices, *p_ijh_*, and quantities, *q_ijh_*, of product (UPC) *i*, belonging to product group *j* by household *h* (in year *t*). This appendix explains how we aggregate these household-level data on individual product prices and quantities (*p_ijh_* and *q_ijh_*) to household-level aggregate prices and quantities for product groups (*P_jh_* and *Q_jh_*) that we use in our main analysis.

The obvious challenge is that the units of quantities differ across products. While expenditures on different products are easily aggregated, neither prices nor quantities are directly comparable across products. To get around this issue, we use quantity-weighted averages of prices and price-weighted averages of quantities.

From here, we proceed in two steps. In the first step, we calculate household-level aggregate quantities for product groups, *Q_jh_*, as weighted sum of the quantities of all products in that group

$$Q_{\mathrm{jh}}=\sum_{i\in j}w_{\mathrm{ijh}}^{P}q_{\mathrm{ijh}}$$ (3)

where weights $w_{\mathrm{ijh}}^{P}$ represent the average price of each product across households

$$Q_{\mathrm{jh}}=\sum_{i\in j}\frac{{\bar{p}}_{\mathrm{ij}}}{{\tilde{P}}_{j}}q_{\mathrm{ijh}}$$ (4)

We normalize all aggregate prices to have an average of one across households (${\tilde{P}}_{j}=1$, ∀ *j*) so that all quantities are expressed as numbers of baskets of all products in a product group. Furthermore, the reason why weights are not household specific is the following. If total expenditures on a product group *j* are different between two households *h* and *h*^′^ only because they paid different prices for one or more products in this group, then *P_jh_* ≠ *P*_*jh*^′^_ and *Q_jh_* = *Q*_*jh*^′^_, whereas if total expenditures on this product group between the two households are different only because they purchased different quantities of one or more products, then *P_jh_* = *P*_*jh*^′^_ and *Q_jh_* ≠ *Q*_*jh*^′^_.

Aggregating prices, *P_jh_*, similarly is not possible because there are many products, of which a household purchases zero quantity, in which case *p_ijh_* is missing in the dataset. To circumvent this problem, in the second step, we start from the expression for total expenditures of household *h* on product category *j*, which must satisfy

$$P_{\mathrm{jh}}Q_{\mathrm{jh}}=\sum_{i\in j}p_{\mathrm{ijh}}q_{\mathrm{ijh}}$$ (5)

From here, it is straightforward to calculate household-level aggregate prices for a product group as the ratio between household-level expenditures and the household-level aggregate quantity for that product group that we obtained in the first step

$$P_{\mathrm{jh}}=\frac{\sum_{i\in j}p_{\mathrm{ijh}}q_{\mathrm{ijh}}}{Q_{\mathrm{jh}}}$$ (6)

### Model

Households are indexed by *i* ∈ *N* and live and shop for food in locations indexed by *j* ∈ *Q*. Households differ from each other in their income *y_ij_*, the distribution of which we treat as exogenous. Households spend an amount *m*(*y_ij_*) of their income *y_ij_* on food. Food expenditure is exogenous and observable and will be taken directly from the data. Households choose how to allocate their total food expenditure between consumption of healthy food *C*_*H*,*ij*_ and unhealthy food *C*_*U*,*ij*_ to maximize their utility function over food

$$u(C_{H,\mathrm{ij}},C_{U,\mathrm{ij}})={\left[\alpha (y_{\mathrm{ij}}){\left(C_{H,\mathrm{ij}}\right)}^{\frac{\varepsilon -1}{\varepsilon }}+(1-\alpha (y_{\mathrm{ij}})){\left(C_{U,\mathrm{ij}}\right)}^{\frac{\varepsilon -1}{\varepsilon }}\right]}^{\frac{\varepsilon }{\varepsilon -1}}$$

The parameter α(*y_ij_*) allows preferences for healthy food to be correlated with income *y_ij_*. This nonhomotheticity of the utility function is the first way in which preferences may affect diets. The only real restriction on the variation of diets with income is that the elasticity of substitution between healthy and unhealthy food ε is assumed to be constant across households of different income levels. The restriction is necessary for identification (see below).

Consumers maximize their utility *u*(*C*_*H*,*ij*_, *C*_*U*,*ij*_) subject to a standard budget constraint

$$P_{H,\mathrm{ij}}C_{H,\mathrm{ij}}+P_{U,\mathrm{ij}}C_{U,\mathrm{ij}}=m(y_{\mathrm{ij}})$$ (7)

where *P*_*H*,*ij*_ and *P*_*U*,*ij*_ are the prices of healthy and unhealthy food, which the household takes as given. These prices may vary across households not only to reflect that different households live and shop in different locations *j* with potentially different price levels but also to allow the possibility that richer households will buy food of higher quality. This is the second way in which preferences may affect diet.

The first-order conditions give the relative consumption of healthy over unhealthy food as a function of preference parameters and prices

$$\frac{C_{H,ij}}{C_{U,\mathrm{ij}}}={\left[\frac{1-\alpha (y_{\mathrm{ij}})}{\alpha (y_{\mathrm{ij}})}\frac{P_{H,\mathrm{ij}}}{P_{U,\mathrm{ij}}}\right]}^{-\varepsilon }$$ (8)

This is a (relative) demand equation, where ε is the price elasticity of demand. A household consumes a healthier diet if their preference for healthy food α(*y_ij_*) is stronger or if healthy food is cheaper relative to unhealthy food.

Food, both healthy and unhealthy, is supplied by retailers. Retailers buy food from a wholesaler so that the marginal cost of healthy and unhealthy food of the same quality is the same function of income in all locations, i.e., *κ_Z_*(*y_ij_*) does not depend directly on *j* conditional on *y_ij_*. However, the marginal costs differ with the quality of the food. To the extent that some households purchase higher quality food than others, the marginal cost of supplying this food will be higher too. We model this by assuming that the marginal costs in the two sectors, denoted by *κ_H_*(*y_ij_*) and *κ_U_*(*y_ij_*), vary exogenously with household income *y_ij_*.

In addition to their marginal costs, retailers face a fixed cost of supplying healthy food *K_H_* or unhealthy food *K_U_* in a particular location *j*. These fixed costs are allowed to vary across locations so that $K_{H}({\bar{y}}_{j})$ and $K_{U}({\bar{y}}_{j})$ may depend exogenously on the average income level of household in the location ${\bar{y}}_{j}$.

We assume that there is free entry of food stores in each location. The fixed costs generate increasing returns to food supply so that in equilibrium there will be a single store for each food type in each location. However, the threat of entry implies that the local monopoly is only sustainable if retailers make zero profits.

A local sustainable monopoly with zero profits implies that prices are set just high enough to recover fixed costs, which leads to the following pricing conditions

$$P_{H,\mathrm{ij}}=\frac{K_{H}({\bar{y}}_{j})}{{\bar{C}}_{H,j}}+\kappa_{H}(y_{\mathrm{ij}})$$ (9)

$$P_{U,\mathrm{ij}}=\frac{K_{U}({\bar{y}}_{j})}{{\bar{C}}_{U,j}}+\kappa_{U}(y_{\mathrm{ij}})$$ (10)

where ${\bar{C}}_{H,j}=\sum_{i\in j}C_{H,\mathrm{ij}}$ and ${\bar{C}}_{U,j}=\sum_{i\in j}C_{U,\mathrm{ij}}$ denote the total consumption in location *j* of healthy and unhealthy food, respectively. Note that these pricing equations imply a downward-sloping supply curve: The more food is sold, the lower the price the retailer will charge for it.The equilibrium of our model is defined as follows.

**Definition:** An equilibrium in our model is an allocation of quantities {*C*_*H*,*ij*_, *C*_*U*,*ij*_} and a set of prices {*P*_*H*,*ij*_, *P*_*U*,*ij*_}, ∀*i* ∈ *N* and *j* ∈ *K*, such that, for a given distribution of income over households and locations, *y_ij_* for all *i* ∈ *N* and *j* ∈ *K*:

1.all households *i* maximize utility subject to their budget constraints so that Eqs. 7 and 8 are satisfied ∀*i*;

2.all retailers in both sectors set prices satisfying Eqs. 3 and 4 ∀*i*, *k*;

3. aggregation identities ${\bar{C}}_{H,j}=\sum_{i\in j}C_{H,\mathrm{ij}}$ and ${\bar{C}}_{U,j}=\sum_{i\in j}C_{U,\mathrm{ij}}$ are satisfied for all locations *j* ∈ *K*.

Hence, the equilibrium is a well-defined system of 4×*N* nonlinear equations in 4×*N* unknowns.

Two observations are important for future reference. First, the fixed costs associated with food supply introduce an externality: The more food of a certain type a household purchases, the cheaper this type of food becomes for other households in the same location that shop in the same store. This externality is the distortion in the food environment that will generate inefficiencies in equilibrium diets. Second, although prices are household specific, only aggregate demand affects prices (by lowering the impact of fixed costs on the average cost per unit sold). This property of the model will be key to our identification strategy, as explained below.

### Identification strategy

Taking within-location deviations of the supply (Eqs. 3 and 4) gives ${\hat{P}}_{H,\mathrm{ij}}={\hat{\kappa }}_{H}(y_{\mathrm{ij}})$ and ${\hat{P}}_{U,\mathrm{ij}}={\hat{\kappa }}_{U}(y_{\mathrm{ij}})$, where a hat over a variable denotes within-location deviations, i.e., ${\hat{X}}_{\mathrm{ij}}=X_{\mathrm{ij}}-{\bar{X}}_{j}$. These equations immediately identify the income dependence of the variable costs or preferences over quality. Moreover, since variation in prices across households within locations is exogenous to household-level demand, we can identify the relative demand equation in within-location deviations from an ordinary least-squares regression of the log of relative quantities of healthy over unhealthy food on the log of relative prices and income

$$\hat{\text{log}(\frac{C_{H,\mathrm{ij}}}{C_{U,\mathrm{ij}}})}=-\varepsilon \hat{\text{log}(\frac{P_{H,\mathrm{ij}}}{P_{U,\mathrm{ij}}})}-\varepsilon \hat{\text{log}\;(\frac{1-\alpha (y_{\mathrm{ij}})}{\alpha (y_{\mathrm{ij}})})}$$

This regression identifies the elasticity of substitution between healthy and unhealthy food ε, as well as the income dependence of preferences for healthy food $\hat{\alpha }(y_{\mathrm{ij}})$. Notice that this would not be the case if we were using more generic preferences in which the elasticity of substitution was a function of income, as in (*23*).

The supply and demand system described by Eqs. 7, 8, 3, and 4 implies a reduced-form relation of consumption of healthy food *C*_*H*, *ij*_, consumption of unhealthy food *C*_*U*,*ij*_, and prices *P*_*H*,*ij*_ and *P*_*U*,*ij*_ of healthy and unhealthy food, respectively, with income. These relations depend on the income dependence of preferences $\alpha ({\bar{y}}_{j})$, marginal costs $\kappa_{H}({\bar{y}}_{j})$ and $\kappa_{U}({\bar{y}}_{j})$, and fixed costs $K_{H}({\bar{y}}_{j})$ and $K_{U}({\bar{y}}_{j})$. Since $\alpha ({\bar{y}}_{j})$, $\kappa_{H}({\bar{y}}_{j})$, and $\kappa_{U}({\bar{y}}_{j})$ follow directly from the within-location estimates of α(*y_ij_*), *κ_H_*(*y_ij_*), and *κ_U_*(*y_ij_*), the estimated reduced form allows us to identify the level and the income dependence of the fixed costs.

### Estimation procedure

We implement the estimation of the model through nonlinear simulated method of moments. The moments are the distance between model and data for *C*_*H*,*ij*_, *C*_*U*,*ij*_, *P*_*H*,*ij*_, and *P*_*U*,*ij*_ for all levels of income. We summarize the information in the data as the average value of these variables in 5-percentile bins for income, both across households in deviation from the location average and across locations. This gives 160 moments to match (20 income quantiles for four variables, each both within and across locations). To facilitate efficient estimation, we approximate the income dependence of the parameters α(*y*), *κ_H_*(*y*), *κ_U_*(*y*), *K_H_*(*y*), and *K_U_*(*y*) as fourth-order polynomials, which gives us a total of 26 parameters to estimate.

Our estimator finds the model parameters that minimize the distance between model and data

$$\hat{\Theta }=\text{arg}\;\underset{\Theta }{\text{min}}{\left[M(\Theta ,Y)-M(Y)\right]}^{\prime }D[M(\Theta ,Y)-M(Y)]$$

Here, *M*(*Y*) is a column vector containing our 160 moments in the data, which are a function of the observed distribution of income within and across location, denoted by *Y*, and *M*(Θ, *Y*) denotes their model counterpart, which is a function of the parameters to be estimated, gathered in the vector Θ.

The weighting matrix *D* is calculated directly from the microdata. It is a diagonal matrix of dimension 160, with diagonal element *d_ii_* equal to the inverse of the standard error of moment *i*, for *i* = 1, …,160. By calculating these standard errors directly from the microdata, we solve the problem that different moments are in different units. Since each moment is an average of a variable over a group of households in the microdata, the standard error of a moment is simply the square root of the variance of that variable divided by the number of households in that group.

### Welfare analysis

In this appendix, we describe the technical steps for the welfare analysis. The first step is computing the relative consumption of fruits and vegetables in the first-best allocation. A social planner chooses the allocation of healthy and unhealthy food for all consumers to maximize the weighted sum of their utility as in the utility function above, using Pareto weights θ*_ij_* to compare utility across consumers

$$\underset{{\left\{C_{H,\mathrm{ij}},C_{U,\mathrm{ij}}\right\}}_{i\in N}}{\text{max}}\sum_{i\in N}\theta_{\mathrm{ij}}u(C_{H,\mathrm{ij}},C_{U,\mathrm{ij}})$$

Retailers make zero profits in equilibrium so that we do not have to worry about how profits are redistributed to consumers. The planner is constrained by the retail technology, the distribution of consumers *i* ∈ *N* over locations *j* ∈ *K*, and the total amount of food expenditure *m*(*y_ij_*), and must respect the aggregate resource constraint. Technically, we also constrain the planner to keep both food markets open in all locations, because otherwise the planner would trivially choose to centralize the production of each type of food in a single location and pay the fixed costs only once

$$\sum_{i\in N}[\kappa_{H}(y_{\mathrm{ij}})C_{H,\mathrm{ij}}+\kappa_{U}(y_{\mathrm{ij}})C_{U,\mathrm{ij}}]+\sum_{j\in K}[K_{H}({\bar{y}}_{j})+K_{U}({\bar{y}}_{j})]=\sum_{i\in N}m(y_{\mathrm{ij}})$$ (11)

In the efficient allocation, the relative consumption of healthy food depends only on preferences over diet and marginal costs, which reflect preferences over quality of food

$$\frac{C_{H,\mathrm{ij}}}{C_{U,\mathrm{ij}}}={\left[\frac{1-\alpha (y_{\mathrm{ij}})}{\alpha (y_{\mathrm{ij}})}\frac{\kappa_{H}(y_{\mathrm{ij}})}{\kappa_{U}(y_{\mathrm{ij}})}\right]}^{-\varepsilon }$$ (12)

Notice that the efficient relative consumption, and therefore the distortions, does not depend on the Pareto weights.

The efficient allocation may also differ from the equilibrium in the distribution of food over consumers: The social planner may want to redistribute food with respect to the equilibrium consumption allocation. The efficient amount of redistribution will depend crucially on the planner’s Pareto weights. For reasons of exposition, we will start with the case of equal Pareto weights, θ*_ij_* = 1 for all *i* ∈ *N*, which will involve a good amount of redistribution. However, we will then consider the case where Pareto weights are chosen such that there is no redistribution at all. We do this to focus on the distortions in relative food prices, in search of a government intervention that knows no losers but only winners. The question what is the optimal amount of redistribution is unrelated to the food environment and therefore outside the scope of this paper.

The second step is then computing the efficient allocation without redistribution. To this goal, we have to take a stance on what “no redistribution” means. We define the “no-redistribution efficient allocation” by imposing two additional constraints on the social planner’s problem. First, the planner does not move resources across locations so that the fixed costs of retailing healthy and unhealthy food in each location must be paid for by consumers in that location. Second, the planner must fund the fixed costs by levying a proportional tax τ on equilibrium total food expenditures *m_ij_*.

The combined constraints on the planner’s problem can be represented by replacing the aggregate resource constraint (Eq. 11) by a resource constraint and a “planner budget constraint” for each location *j*

$$\sum_{i\in j}[\kappa_{H}(y_{\mathrm{ij}})C_{H,\mathrm{ij}}+\kappa_{U}(y_{\mathrm{ij}})C_{U,\mathrm{ij}}]=\sum_{i\in j}(1-\tau_{j})m(y_{\mathrm{ij}})$$ (13)

$$K_{H}({\bar{y}}_{j})+K_{U}({\bar{y}}_{j})=\sum_{i\in j}\tau_{j}m(y_{\mathrm{ij}}),\;\;\forall j\in Q,$$ (14)

where the planner chooses τ*_j_* for all locations *j* in addition to the consumption allocation. The constrained efficient allocation subject to these constraints corresponds to the first-best allocation with appropriate Pareto weights to prevent redistribution. The additional constraints do not affect efficiency condition (Eq. 12) for the relative consumption of healthy and unhealthy food.

Regarding welfare, with equal Pareto weights, the planner redistributes consumption from richer to poorer households so that poorer households are much better off in the efficient allocation (utility of the poorest 5% of households is higher in the first-best allocation by a consumption equivalent of 20%), but richer households are made worse off, despite the improvement in their diet. The welfare consequences are markedly different when we choose Pareto weights to avoid redistribution. In this case, richer households are unambiguously better off in the first-best allocation, by a consumption equivalent of slightly over 0.25 % , because of their improved diet. However, poorer households suffer a welfare loss from being taxed for the fixed costs of supplying healthy food that they would not have chosen to consume in equilibrium. This is particularly true for poor households living in rich counties, where the fixed costs of supplying fruits and vegetables are higher.

Who are the winners and losers from a policy intervention that eliminates price distortions due to fixed costs in the supply of fruit and vegetables depends on how the planner funds this policy, in particular on how much consumption is redistributed across households. This raises the question whether it is possible to design an intervention to eliminate distortions that make all households better off. It is worth noting that, in the no-redistribution case, the average consumer in all counties is strictly better off in the first-best allocation. Within counties, some consumers are made worse off because for them the utility loss from the higher tax on consumption exceeds the utility gain from a better diet. As a third step, we now explore whether it is possible to compensate these losers through a different tax policy.

Consider a household-specific tax rate τ*_ij_* instead of a location-specific tax τ*_j_* in Eqs. 13 and 14. We look for an income-dependent tax rate τ*_ij_* = τ*_j_*(*y_ij_*) that generates the same welfare gain for all households within a county. The welfare gains from moving to the efficient allocation under this policy are almost constant for all households at 0.1% of consumption, as displayed by the orange diamond line. There are small differences between households that live in different locations, but since we already showed that the average household in each county improves its welfare, now that the welfare gains are equalized within counties, it must be that welfare improves for all households.

Under the Pareto-improving efficient allocation, fruit and vegetable purchases are subsidized, and households are taxed for other food consumption at a rate that slightly increases with their income and varies with the location where they live. The administration costs of this optimal policy would be very high, but it is possible to approximate it with a simple rule that is feasible to implement in reality. This simple rule would subsidize fruits and vegetables by a constant amount across consumers, where this subsidy is funded by a small increase in income taxes to offset the unintentional redistributive effect of the subsidy, as we illustrate in the main text.
